# Thirty-Fifth Annual Meeting of the Michigan State Dental Association

**Published:** 1890-08

**Authors:** 


### Proceedings and Papers

### Of the Thirty-Fifth Annual Meeting of the Michigan State Dental Association.

THIRD DAY.—THURSDAY MORNING, JUNE 5TH.

Association call to order at 8:30 A. M.

The Board of Directors, through President Case, reported that they had examined into the matters of Dr. L. L. Davis, who was charged with a violation of the code of ethics, and found that he was guilty, but recommended that the society deal leniently with him because of his previous good standing in the profession, and that there are circumstances which mitigate the offence.

Dr. Davis was asked for a statement of his side of the case, which he gave, and said he was sorry the thing had ever occurred, and he did not think he would ever do such a thing again.

After considerable discussion on both sides Dr. Davis was asked if he was willing to say that he had violated the code of ethics of the Michigan Dental Association and was sorry for it, and if forgiven would promise never to do so again. He would not subscribe to such a definite statement, and by vote of the association he was suspended for one year.

Dr. J. Ward House, of Grand Rapids, read a paper on "Dental Ethics from the Standpoint of a Young Practitioner."

